# Supplementary material for: Expansion and collapse of VEGF diversity in major clades of the animal kingdom
Source: Angiogenesis. 2023 Apr 5;26(3):437–61. doi: 10.1007/s10456-023-09874-9 (PMC10328876; doi:10.1007/s10456-023-09874-9)
Supplement: Supplementary file 1 — Supplementary file1 (PDF 6158 KB) [file 10456_2023_9874_MOESM1_ESM.pdf]

Supplementary Information

Expansion and collapse of VEGF diversity in major clades of the animal kingdom

Khushbu Rauniyar[1]; Honey Bokharaie[1]; Michael Jeltsch[1,2,3,4,*]

[1]Drug Research Program, Division of Pharmaceutical Biosciences, Faculty of Pharmacy, University of Helsinki, Finland; [2]Individualized Drug Therapy Research Program, Faculty of Medicine, University of Helsinki, Finland; [3]Wihuri Research Institute, Helsinki, Finland, [4]Helsinki One Health, University of Helsinki, Finland

*Author for correspondence: Dr. Michael Jeltsch, Drug Research Program, Faculty of Pharmacy, Biocenter 2, University of Helsinki, P.O.B. 56 (Viikinkaari 5E), 00790 Helsinki, Finland; Phone: +358-2941-25514; Fax: +358-2941-25510; E-mail: michael@jeltsch.org

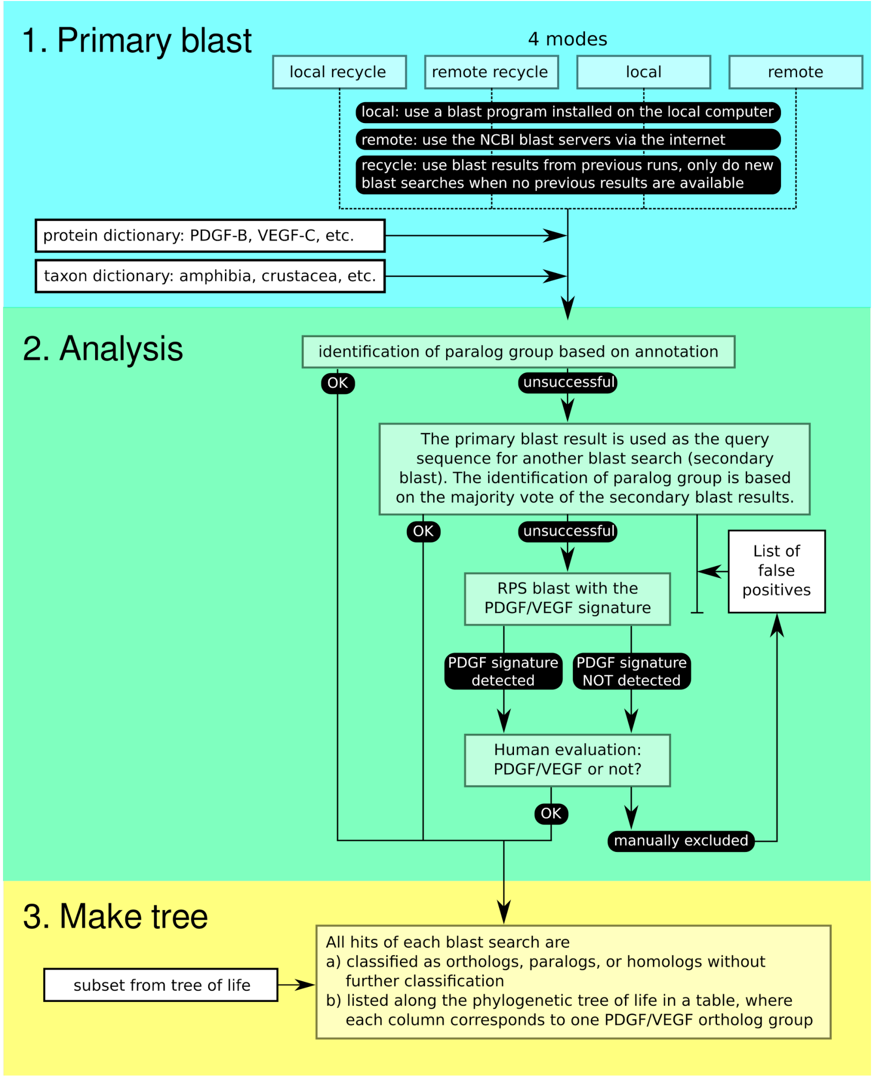

Supplementary Fig. S1 Bioinformatics workflow of the PDGF/VEGF search The workflow resembles crowdsourcing by recursively analyzing the manual and programmatic descriptions of protein database entries most similar to the protein of interest. Only if this method fails, the protein is classified as a "generic" PDGF/VEGF homolog based on the detection of the PDGF signature (https://prosite.expasy.org/PDOC00222).

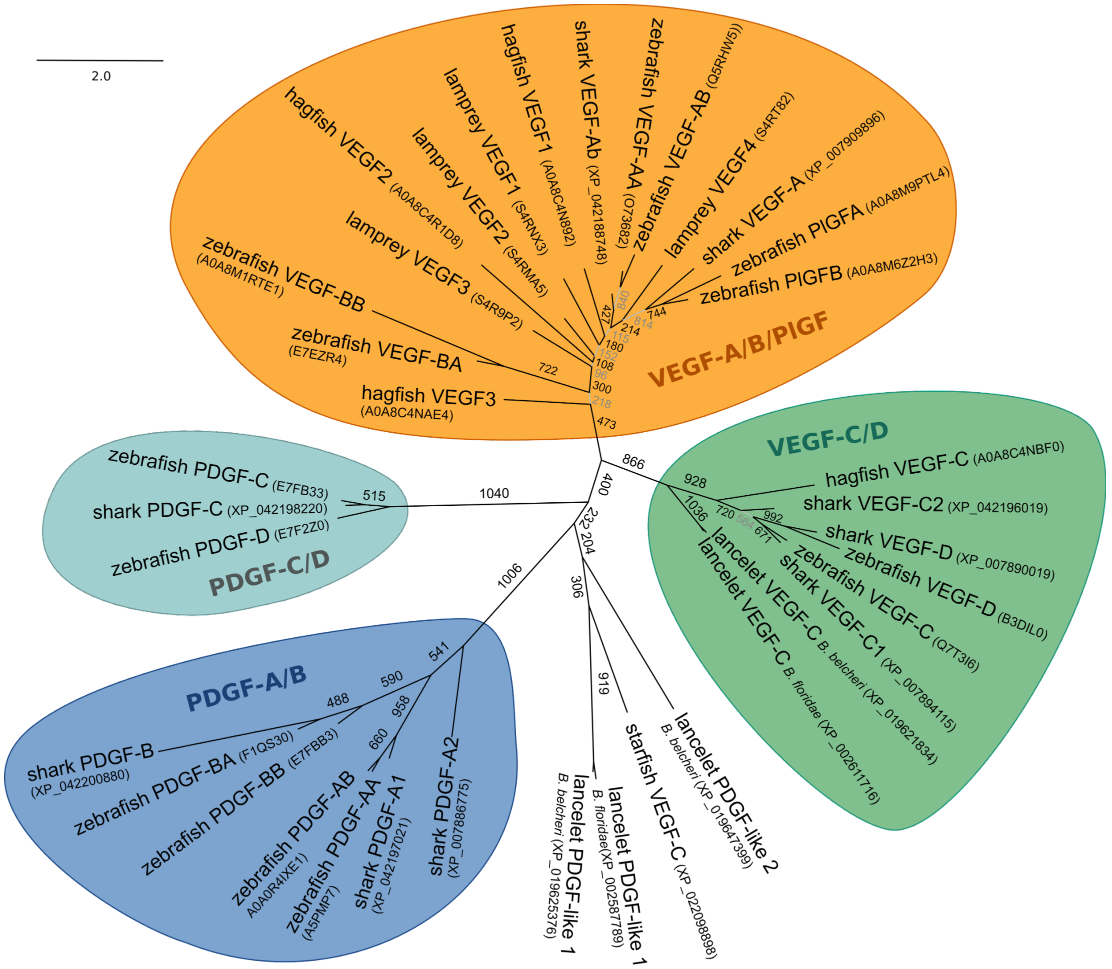

Supplementary Fig. S2 Relationship of early fish PDGFs/VEGFs An unrooted tree of the relationships of PDGFs/VEGFs found in Florida and Chinese lancelets (*Branchiostoma floridae*, *Branchiostoma belcheri*), the inshore hagfish (*Eptatretus burgeri*), the sea lamprey (Petromyzon marinus), the elephant shark (*Callorhinchus milii*) and zebrafish (*Danio rerio*) shows that many of the early vertebrate PDGFS/VEGFs cannot be easily designated as orthologs of any modern VEGF or PDGF. Only with the appearance of bony fish, it becomes consistently possible to identify orthologs of these factors among the nine mammalian PDGFs/VEGFs. While there are clear VEGF-C-like proteins in jawless fish, most of their PDGF/VEGF-like molecules are difficult to classify. In this tree, such PDGFs/VEGFs are shown to be most closely related to VEGF-A/VEGF-B or PlGF, but bootstrap supports are generally weak. Even less clear are the PDGFs/VEGFs of Echinodermata and Cephalochordata, which appear equally distant to all the big groups (PDGFs, VEGF-C/D, VEGF-A/B/PlGF). Therefore, the assignment of PDGFs/VEGFs more distantly related to mammals than bony fish (such as shown in Fig. 4) is hypothetical and relies mostly on the presence (VEGF-C-like) or absence (VEGF-A-like) of multiple BR3P repeats of BR3P motif.

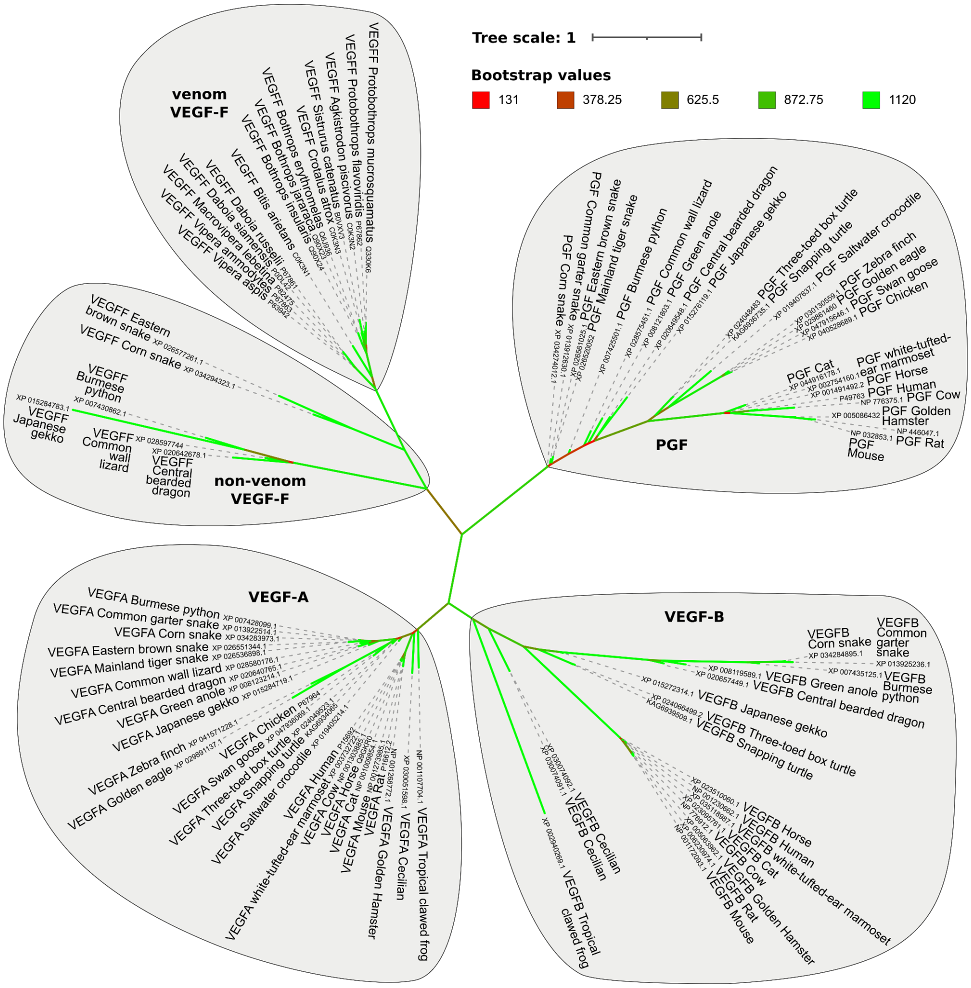

Supplementary Fig. S3 Phylogenetic tree of VEGF-Fs An unrooted tree of the relationship of VEGF-F to the other hemangiogenic VEGFs (VEGF-A, VEGF-B, PlGF). VEGF-Fs segregate into multiple branches, supporting the two-origin hypothesis of venom (once in snakes, once in lizards, [86]). Assuming the prevailing two-origin hypothesis of venom, the independent emergence of VEGF-F as a venom component on multiple branches appears unlikely; even more so since several snake VEGF-Fs segregate with the non-venom ("lizard") sub-branches. While venom VEGF-F expression is specific to the venom gland [86], it is not ubiquitous among venomous snakes and is found only in the viper clade (see also Supplementary Figure S14 in [86]). The most parsimonious explanation is a single origin of VEGF-F, predating the evolution of venom. Its original function is yet to be determined, but a later co-option of the VEGF-F gene in vipers established it as a bonafide venom component. However, in other branches of the tree (lizards and snakes outside the viper clade, such as the non-venomous Burmese python), the VEGF-F gene might serve its unknown original function, assumed a function as a venom component, or has disappeared. The most likely origin of the VEGF-F gene is a VEGF-A gene duplication. An origin via a PGF or VEGF-B gene duplication is less likely, but not impossible.

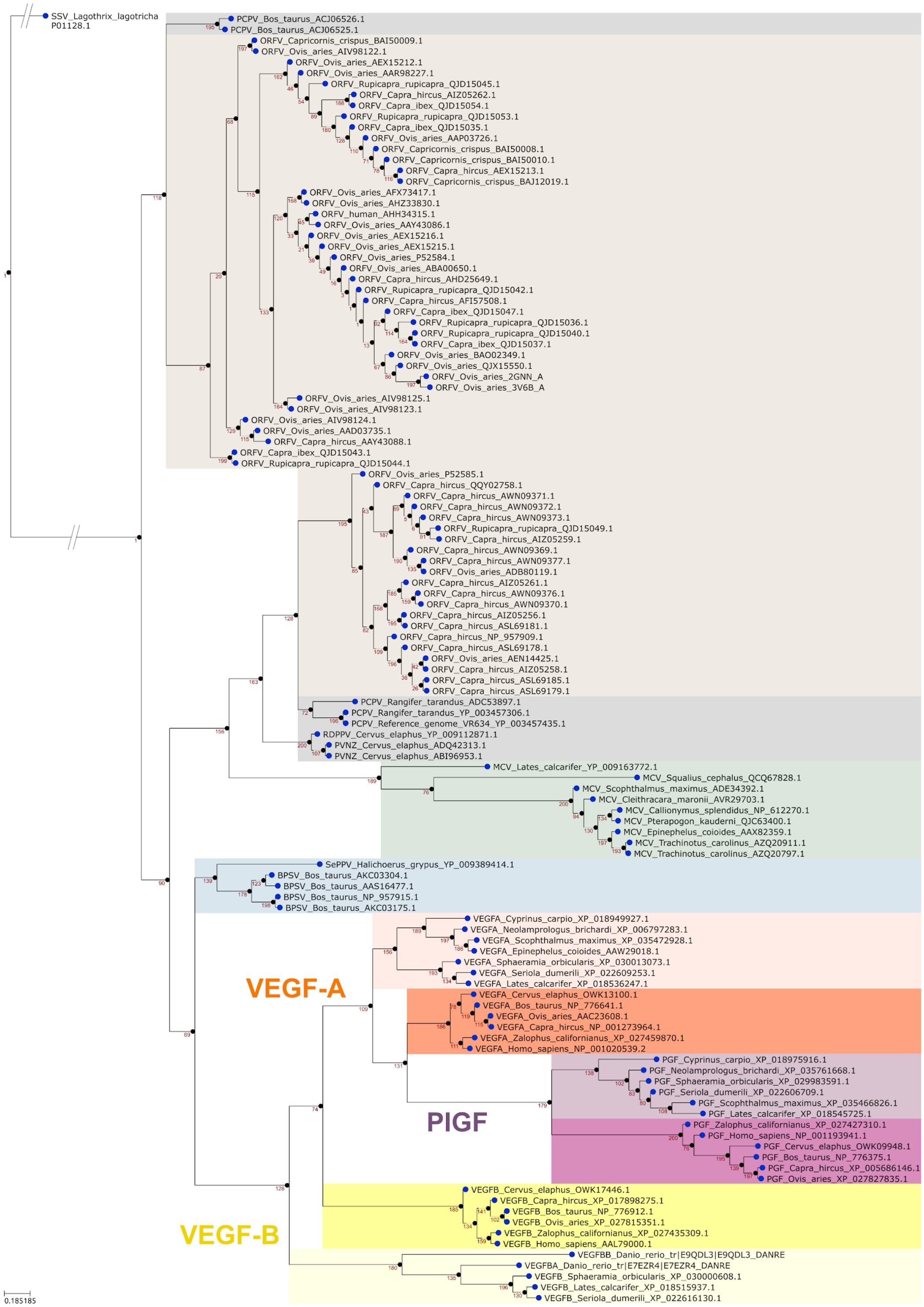

Supplementary Fig. S4 Uncollapsed phylogenetic tree of the VEGF-E tree in Fig.7 The most likely candidate for the VEGF-E origin is a single VEGF-A gene acquisition event from a vertebrate host.

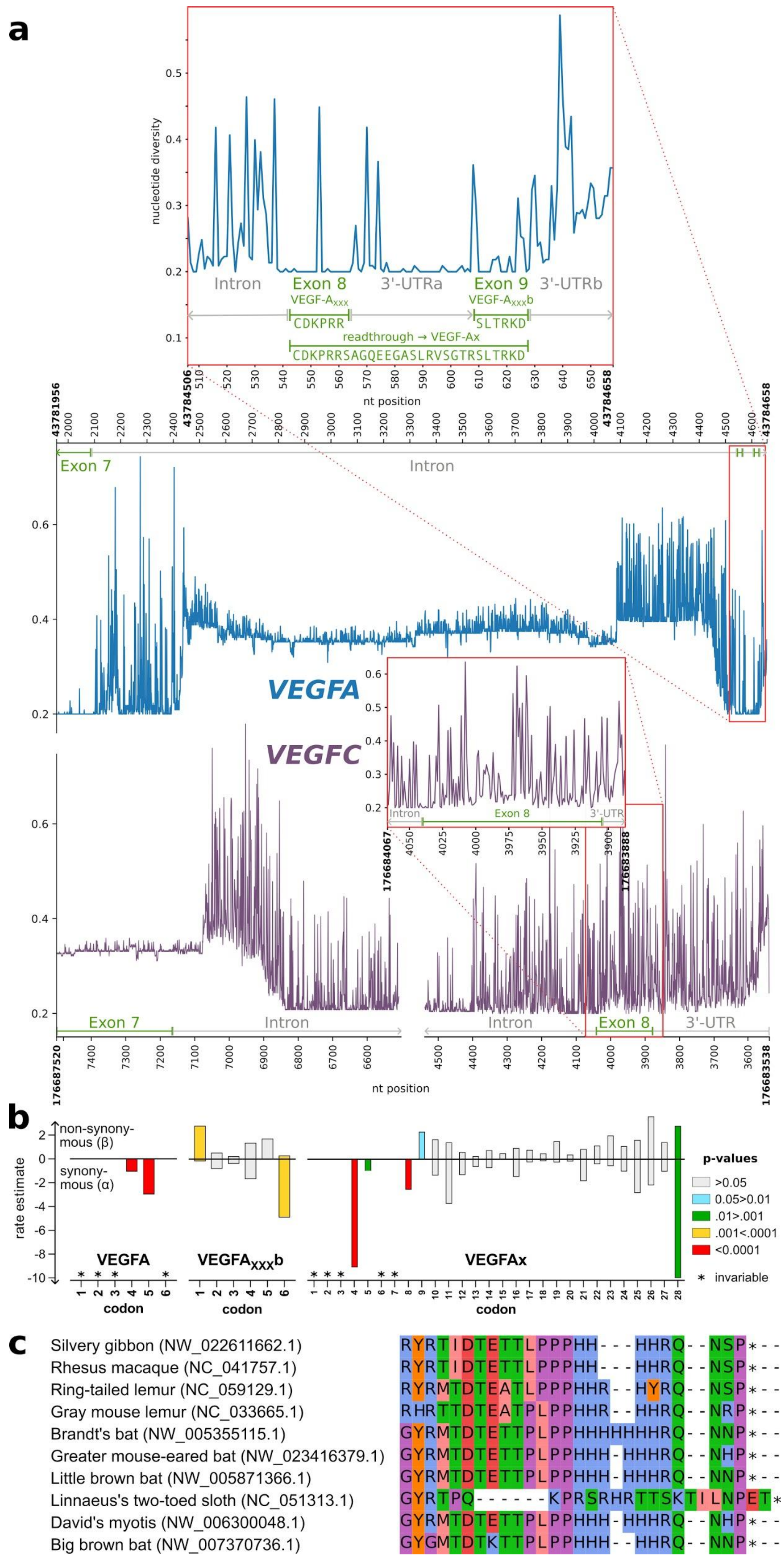
a
nucleotide diversity
Intron
Exon 8
3'-UTRa
Exon 9
3'-UTRb
CDKPRR
SLTRKD
readthrough → VEGF-Ax
CDKPRRSAGQEEGASLRVSGTRSLTRKD
nt position
Exon 7
Intron
VEGFA
VEGFC
Exon 8
3'-UTR
b
non-synonymous (β)
synonymous (α)
rate estimate
VEGFA
VEGFAxxxb
VEGFAx
codon
p-values
>0.05
0.05>0.01
.01>.001
.001<.0001
<0.0001
* invariable
c
Silvery gibbon (NW_022611662.1)
Rhesus macaque (NC_041757.1)
Ring-tailed lemur (NC_059129.1)
Gray mouse lemur (NC_033665.1)
Brandt's bat (NW_005355115.1)
Greater mouse-eared bat (NW_023416379.1)
Little brown bat (NW_005871366.1)
Linnaeus's two-toed sloth (NC_051313.1)
David's myotis (NW_006300048.1)
Big brown bat (NW_007370736.1)

Supplementary Fig. S5 Diversity and conservation of the 3'-end of the human *VEGFA* gene. Panel a shows the diversity of nucleotides 43781956-43784658 of human chromosome 6 (genome assembly GRCh38:CM000668.2) as measured by DIVAA. The magnification of the protein-coding region of exon 8 shows high conservation at the nucleotide level. (b) Analysis of the synonymous (α) versus non-synonymous (β) mutation rate reveals that there is some evidence for purifying selection of all three variations of the VEGF-A C-terminus, with the overwhelmingly strongest evidence for the canonical exon 8. The only amino acid subject to diversifying selection is the first amino acid of exon 9, which overlaps with the consensus sequence for splicing and is thus subject to additional selection pressure at the nucleotide level. (c) In about 10% of the mammals analyzed, the putative exon 9 coding sequence would not terminate after 6 amino acids but continue to encode a longer, predominantly basic tail with a polyhistidine stretch.

Exon numbering is according to the canonical isoform (P15692-1), and the term "exon 9" is used according to Bates et al. [69]. Please note that the heterogenous diversity within the introns is due to indels specific to certain animal clades, caused by transposition events.

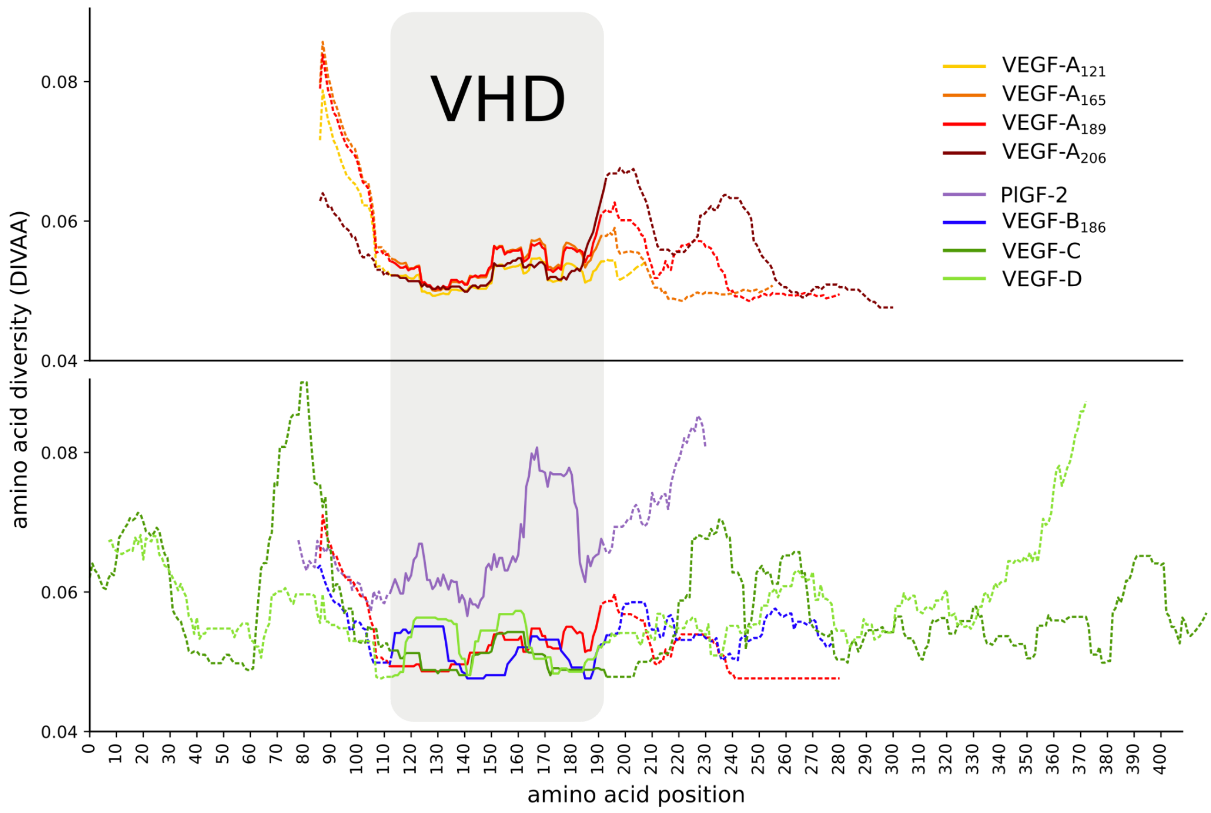

Supplementary Fig. S6 DIVAA analysis of VEGF-A isoforms (top panel) and the other mammalian VEGF family members (bottom panel) Although VEGF-A_{165} is the major isoform in humans, database entry frequencies indicate that VEGF-A_{189} might be the predominant VEGF-A isoform in many species. The diversity of all VEGFs is lowest in the VHD (i.e., the receptor binding domain) and increases both N- and C-terminally. From the VEGF family members, PlGF-2 shows the highest and VEGF-C the lowest level of diversity in the VHD.

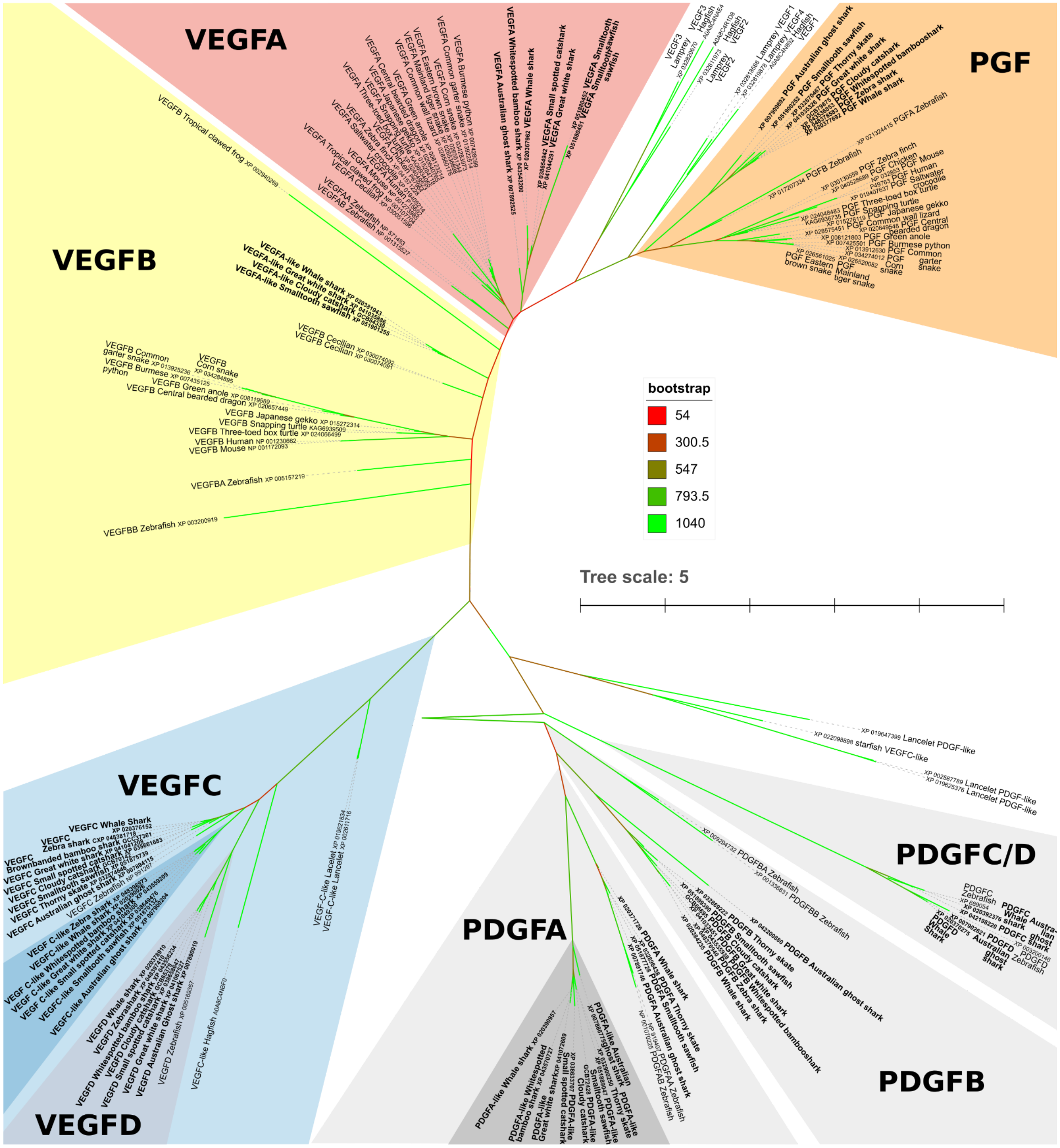

Supplementary Fig. S7 Sharks feature PlGFs and VEGF-B, and duplicated *vegfc* and *pdgfa* genes Cartilaginous fish (chondrichthyes) arguably feature already orthologs to all nine mammalian PDGFs/VEGFs. However, since the separation of VEGF-A, VEGF-B and PlGF had been a fairly recent event when chondrichthyes separated from bony fishes, a clean assignment of individual genes into the VEGF-B versus the VEGF-A paralog group is ambiguous (these genes are labeled as VEGF-A-like in protein databases). Like zebrafish VEGF-B, these genes do not produce two transcripts with overlapping reading frames characteristic of mammalian VEGF-B. The C-termini of the translated proteins are highly positively charged similar to the

human VEGF-B_{167} and VEGF-A_{165} isoforms. Chondrichthyes' VEGF-A genes are often labeled as VEGF-Ab or VEGF-Ab-like, even though cartilaginous fish - different from zebrafish - did not undergo the teleost whole genome duplication. Interestingly, all sharks analyzed featured a duplicated VEGF-C and PDGF-A gene ("VEGFC-like" and "PDGFA-like").

PDGFs/VEGFs from cartilaginous fishes are shown in bold. For VEGF-C, VEGF-D, and PDGFs, the corresponding tetrapod proteins are not shown. With one exception, lancelet and cyclostomate proteins are not assigned to ortholog groups due to the low confidence in the branching. Confidence values were determined by bootstrap analysis and are shown in a color scale from red (low confidence) to green (high confidence).

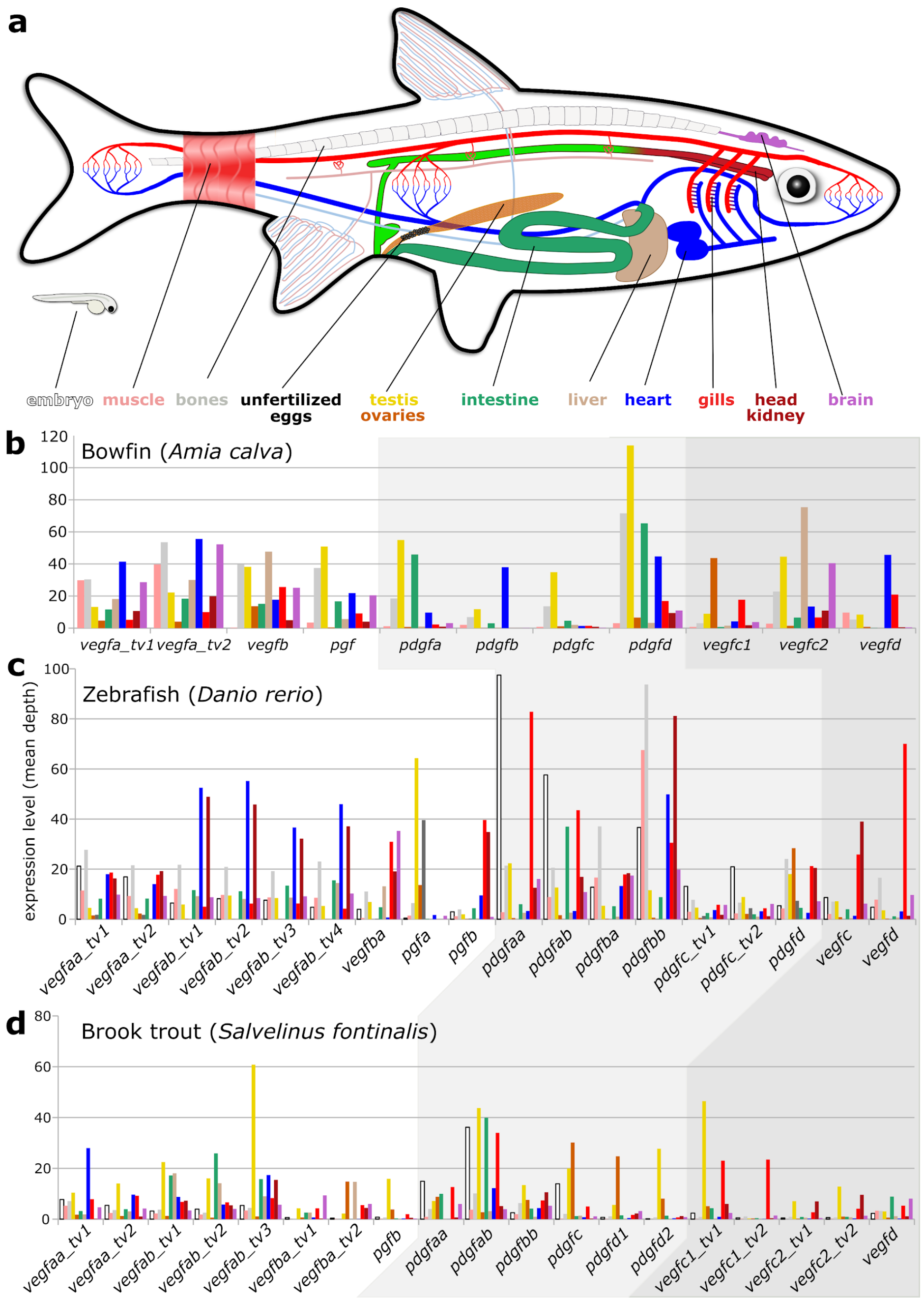

Supplementary Fig. S8 Expression of PDGFs and VEGFs in bowfin, zebrafish, and brook trout The bowfin has undergone none, zebrafish one, and brook trout two whole genome duplications after the second common vertebrate genome duplication. The analyzed tissues are color-coded, as shown in (a). The _tv# extension designates different transcript variants (e.g., splice isoforms). Genes that have been duplicated independently from the teleost genome duplication have been indicated by adding a numeral after the gene name.

Supplementary Table 1. Complete table of all BLAST hits.

Supplementary Table 2. False-positive BLAST hits of PDGF/VEGF-like sequences from the phylum Porifera.

Supplementary Table 3. Fish PDGF/VEGF mRNA transcript contigs.

Supplementary Table 4. Complete list of all 24 fish mRNA data.
