# Supplementary material for: Expansion and collapse of VEGF diversity in major clades of the animal kingdom
Source: Angiogenesis. 2023 Apr 5;26(3):437–61. doi: 10.1007/s10456-023-09874-9 (PMC10328876; doi:10.1007/s10456-023-09874-9)
Supplement: Supplementary file 3 — Supplementary table1 (PDF 147 KB) [file 10456_2023_9874_MOESM3_ESM.pdf]

- Analysis performed: 191115_161233
- Analyzed sequences (hits resulting from 676 blast searches, 52 animal groups x 13 query sequences):49992 (out of which unique: 8666, programmatically recognized as VEGF/PDGF family members: 90.5%).
- Red dotted lines in the tree indicate paraphyletic relationships.
- The tree background color indicates the presence of the proteins with the corresponding color according to our hypotheses.
- The red-to-white background of the table indicates a heuristic reliability of the results, where a brighter color indicates a higher reliability. This is calculated using the number of fully sequenced genomes, the number of species in the phylum and the number of protein sequences available for that phylum.
- The numbers in the table denote the number of: orthologs found (black), P = paralogs found, ? = homologs found, whose relationship could not be programmatically determined, Σ = total homologs found.
- Small (but over time increasing) deviations in the numbers with the dynamically updated online version and the numbers in the manuscript text are normal due to the constantly increasing number of available sequences.

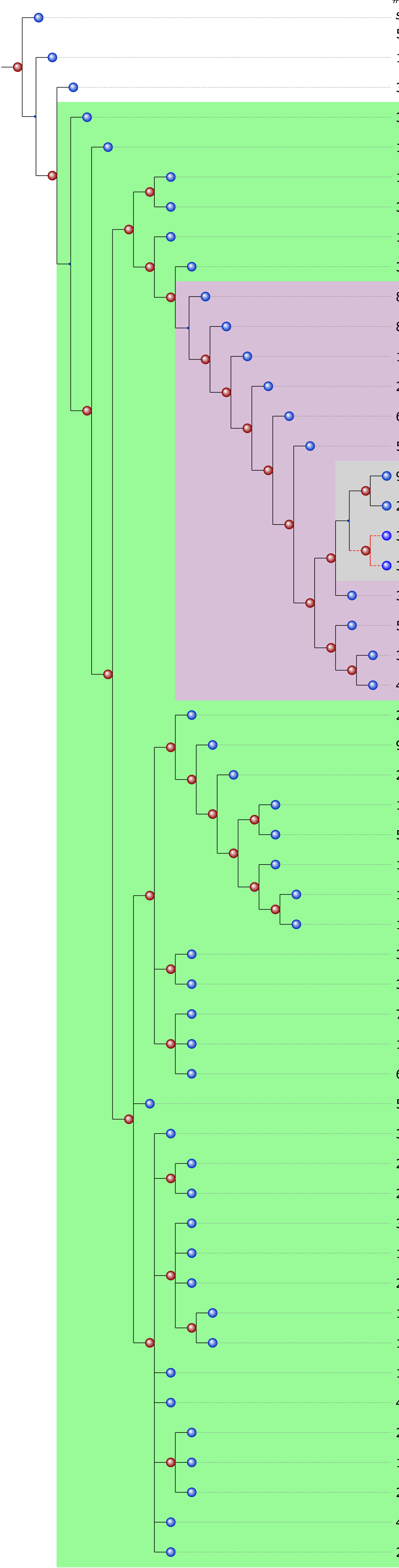

# animal species	# sequences	# compl. genomes	# unique blasthits (excl. false pos.)		PDGF-A	PDGF-B	PDGF-C	PDGF-D	PlGF-1
55	6	3	0 (0)	ctenophora (comb jellies)					
1388	34k	2	11 (0)	porifera (sponges)					
3	36k	2	0 (0)	placozoa					
3730	115k	18	94 (72)	cnidaria (medusae/polyps)	0 P6, ?4, Σ10	1 P3, ?1, Σ5	0 P1, ?0, Σ1		0 P2, ?1, Σ3
151	925	0	1 (0)	xenacoelomorpha					
1803	136k	11	42 (40)	echinodermata	0 P5, ?1, Σ6	0 P5, ?0, Σ5	0 P11, ?1, Σ12	0 P12, ?1, Σ13	0 P9, ?3, Σ12
39	23k	2	9 (6)	hemichordata (acorn wormws)	0 P2, ?0, Σ2	0 P2, ?0, Σ2	0 P2, ?1, Σ3	0 P2, ?0, Σ2	0 P1, ?0, Σ1
11	95k	4	20 (14)	cephalochordata (lancelets)	0 P6, ?1, Σ7	0 P6, ?1, Σ7	0 P5, ?1, Σ6	0 P5, ?1, Σ6	0 P5, ?0, Σ5
363	64k	6	2 (1)	tunicata	0 P1, ?0, Σ1	0 P1, ?0, Σ1			0 P1, ?1, Σ2
80	8k	3	0 (0)	cyclostomata (hagfish/lamprey)					
844	115k	6	52 (51)	chondrichthyes (cartilaginous fishes)	6 P21, ?0, Σ27	2 P25, ?0, Σ27	2 P18, ?0, Σ20	2 P11, ?0, Σ13	0 P25, ?0, Σ25
19326	2M	186	2134 (n.a.)	actinopterygii (ray-finned fishes)	217 P665, ?170, Σ1052	27 P863, ?170, Σ1060	117 P282, ?15, Σ414	148 P269, ?9, Σ426	102 P882, ?159, Σ1143
2	35k	1	31 (30)	coelacanthimorpha (lobe-finned fishes)	1 P15, ?0, Σ16	2 P12, ?0, Σ14	1 P12, ?0, Σ13	2 P4, ?0, Σ6	2 P12, ?0, Σ14
6	1k	0	10 (8)	dipnoi (lungfishes)	1 P3, ?0, Σ4	1 P3, ?0, Σ4	0 P4, ?0, Σ4	0 P4, ?0, Σ4	0 P4, ?0, Σ4
5762	478k	6	144 (144)	amphibia	16 P59, ?1, Σ76	12 P62, ?1, Σ75	6 P54, ?0, Σ60	5 P17, ?0, Σ22	0 P66, ?1, Σ67
9484	3M	132	1551 (n.a.)	aves (birds)	127 P340, ?5, Σ472	118 P461, ?5, Σ584	98 P452, ?2, Σ552	154 P316, ?0, Σ470	109 P463, ?4, Σ576
24	179k	4	102 (102)	crocodylia (crocodiles)	5 P26, ?0, Σ31	1 P14, ?0, Σ15	4 P35, ?0, Σ39	6 P21, ?0, Σ27	7 P46, ?0, Σ53
3282	96k	6	66 (64)	lepidosauria excl. toxicofera (non-poisonous lizards)	3 P52, ?0, Σ55	6 P45, ?0, Σ51	3 P38, ?1, Σ42	5 P18, ?0, Σ23	6 P46, ?1, Σ53
3854	467k	15	161 (155)	toxicofera (poisonous reptiles)	12 P122, ?0, Σ134	9 P113, ?0, Σ122	9 P89, ?0, Σ98	11 P26, ?0, Σ37	14 P91, ?28, Σ133
358	184k	10	188 (179)	testudines (turtles)	9 P65, ?1, Σ75	8 P66, ?1, Σ75	7 P60, ?7, Σ74	17 P20, ?0, Σ37	10 P61, ?1, Σ72
5	26k	1	25 (25)	monotremata (egg-laying mammals)	1 P6, ?0, Σ7	1 P5, ?0, Σ6	3 P10, ?0, Σ13	3 P10, ?0, Σ13	1 P5, ?0, Σ6
335	142k	5	88 (88)	metatheria (marsupials)	7 P36, ?0, Σ43	5 P36, ?0, Σ41	4 P33, ?0, Σ37	10 P16, ?0, Σ26	4 P25, ?0, Σ29
4806	8M	181	2995 (n.a.)	eutheria (placentals)	247 P762, ?6, Σ1015	223 P892, ?13, Σ1128	218 P659, ?1, Σ878	235 P417, ?0, Σ652	261 P1262, ?9, Σ1532
206	46k	2	9 (9)	tardigrada (water bears)	0 P1, ?3, Σ4	0 P3, ?1, Σ4	0 P2, ?0, Σ2	0 P1, ?0, Σ1	0 P2, ?0, Σ2
96	2k	1	0 (0)	onychophora (velvet worms)					
224	2k	0	0 (0)	pycnogonida (sea spiders)					
10419	646k	27	103 (101)	arachnida (spiders)	2 P20, ?3, Σ25	0 P20, ?3, Σ23	0 P21, ?5, Σ26	0 P9, ?2, Σ11	2 P24, ?39, Σ65
5	39k	1	41 (31)	xiphosura (horseshoe crabs)	0 P6, ?3, Σ9	0 P6, ?1, Σ7	0 P7, ?3, Σ10	0 P3, ?2, Σ5	0 P7, ?20, Σ27
1004	7k	1	1 (0)	myriapoda (millipeds)					
11193	947k	25	53 (52)	crustacea	0 P10, ?8, Σ18	0 P11, ?4, Σ15	1 P4, ?3, Σ8	0 P1, ?8, Σ9	0 P5, ?7, Σ12
117155	7M	339	618 (n.a.)	hexapoda (insects)	20 P77, ?117, Σ214	3 P89, ?113, Σ205	1 P42, ?34, Σ77	2 P23, ?16, Σ41	3 P52, ?141, Σ196
30	368	0	0 (0)	nematomorpha (horsehair worms)					
3574	2M	100	44 (44)	nematoda (roundworms)	0 P0, ?35, Σ35	0 P0, ?11, Σ11			
7	21k	1	8 (1)	priapulida (penis worms)	1 P0, ?0, Σ1	0 P1, ?7, Σ8			
1	1	0	0 (0)	loricifera					
62	436	0	0 (0)	kinorhyncha (mud dragons)					
56	2k	0	0 (0)	chaetognatha (arrow worms)					
330	3k	0	1 (0)	bryozoa (moss animals)					
26	155	0	2 (0)	entoprocta					
2	278	0	0 (0)	cycliophora (symbion)					
3435	129k	5	8 (7)	annelida (segmented worms)	0 P0, ?3, Σ3	0 P2, ?2, Σ4	0 P1, ?1, Σ2	0 P1, ?1, Σ2	0 P1, ?2, Σ3
14373	742k	26	38 (34)	mollusca	0 P4, ?6, Σ10	0 P8, ?0, Σ8	0 P4, ?4, Σ8	0 P1, ?4, Σ5	0 P2, ?1, Σ3
262	5k	1	0 (0)	nemertea (ribbon worms)					
100	42k	1	6 (2)	brachiopoda (lamp shells)	0 P1, ?0, Σ1	0 P1, ?0, Σ1	0 P0, ?1, Σ1	0 P1, ?0, Σ1	
14	165	1	0 (0)	phoroniformea (horseshoe worms)					
130	389	0	0 (0)	gastrotricha (hairybacks)					
4537	561k	26	1 (0)	platyhelminthes (flatworms)					
21	79	0	0 (0)	gnathostomulida (jaw worms)					
1	2	0	0 (0)	micrognathozoa					
245	64k	6	6 (2)	rotifera (wheel animals)	1 P0, ?0, Σ1	0 P1, ?0, Σ1	0 P1, ?1, Σ2		0 P1, ?0, Σ1
4	9k	1	1 (0)	orthonectida					
24	150	0	0 (0)	dicyemida					

	VEGF-A121	VEGF-A165	VEGF-A206	VEGF-B167	VEGF-B186	VEGF-C	VEGF-D	VEGF-F
ctenophora (comb jellies)								
porifera (sponges)						1 P0, ?7, Σ8	0 P1, ?1, Σ2	0 P0, ?3, Σ3
placozoa								
cnidaria (medusae/polyps)	0 P6, ?7, Σ13	1 P7, ?13, Σ21	0 P6, ?9, Σ15	0 P18, ?61, Σ79	0 P6, ?0, Σ6	11 P1, ?43, Σ55	0 P7, ?19, Σ26	0 P3, ?2, Σ5
xenacoelomorpha							0 P0, ?1, Σ1	
echinodermata	3 P2, ?7, Σ12	3 P3, ?11, Σ17	3 P2, ?7, Σ12	0 P10, ?9, Σ19	0 P10, ?8, Σ18	2 P8, ?12, Σ22	1 P9, ?7, Σ17	0 P7, ?3, Σ10
hemichordata (acorn wormws)	0 P1, ?2, Σ3	0 P1, ?1, Σ2	0 P1, ?1, Σ2	0 P2, ?1, Σ3	0 P2, ?2, Σ4	2 P1, ?1, Σ4	0 P3, ?1, Σ4	0 P2, ?0, Σ2
cephalochordata (lancelets)	1 P5, ?1, Σ7	1 P5, ?1, Σ7	1 P5, ?1, Σ7	0 P6, ?2, Σ8	0 P6, ?1, Σ7	6 P1, ?6, Σ13	0 P6, ?1, Σ7	0 P6, ?1, Σ7
tunicata	1 P0, ?0, Σ1	1 P0, ?0, Σ1	1 P0, ?0, Σ1	0 P1, ?0, Σ1	0 P1, ?0, Σ1	0 P1, ?0, Σ1	0 P1, ?0, Σ1	0 P1, ?0, Σ1
cyclostomata (hagfish/lamprey)								
chondrichthyes (cartilaginous fishes)	11 P14, ?0, Σ25	11 P14, ?0, Σ25	11 P13, ?0, Σ24	0 P29, ?0, Σ29	0 P29, ?1, Σ30	6 P23, ?0, Σ29	2 P26, ?0, Σ28	0 P25, ?0, Σ25
actinopterygii (ray-finned fishes)	426 P811, ?209, Σ1446	430 P462, ?137, Σ1029	433 P463, ?130, Σ1026	59 P1041, ?154, Σ1254	58 P1161, ?173, Σ1392	175 P1034, ?141, Σ1350	102 P1012, ?136, Σ1250	30 P1054, ?169, Σ1253
coelacanthimorpha (lobe-finned fishes)	3 P12, ?0, Σ15	3 P11, ?0, Σ14	3 P11, ?0, Σ14	3 P14, ?0, Σ17	3 P14, ?1, Σ18	2 P15, ?0, Σ17	1 P16, ?0, Σ17	0 P14, ?0, Σ14
dipnoi (lungfishes)	2 P2, ?0, Σ4	2 P2, ?0, Σ4		0 P4, ?0, Σ4	0 P4, ?0, Σ4	0 P4, ?0, Σ4	0 P4, ?0, Σ4	0 P4, ?2, Σ6
amphibia	27 P38, ?1, Σ66	27 P30, ?1, Σ58	27 P33, ?1, Σ61	3 P69, ?1, Σ73	3 P73, ?1, Σ77	5 P71, ?1, Σ77	6 P72, ?1, Σ79	0 P68, ?1, Σ69
aves (birds)	140 P390, ?9, Σ539	141 P329, ?5, Σ475	141 P329, ?5, Σ475	0 P672, ?8, Σ680	0 P764, ?8, Σ772	130 P704, ?4, Σ838	96 P707, ?4, Σ807	1 P729, ?8, Σ738
crocodylia (crocodiles)	17 P40, ?0, Σ57	17 P35, ?0, Σ52	17 P29, ?0, Σ46	0 P57, ?0, Σ57	0 P63, ?0, Σ63	9 P54, ?0, Σ63	8 P55, ?0, Σ63	0 P53, ?0, Σ53
lepidosauria excl. toxicofera (non-poisonous lizards)	26 P31, ?1, Σ58	26 P26, ?1, Σ53	26 P22, ?1, Σ49	2 P54, ?0, Σ56	2 P59, ?0, Σ61	7 P56, ?1, Σ64	5 P57, ?0, Σ62	4 P48, ?0, Σ52
toxicofera (poisonous reptiles)	53 P63, ?29, Σ145	53 P43, ?29, Σ125	53 P45, ?28, Σ126	7 P109, ?26, Σ142	7 P120, ?26, Σ153	11 P108, ?26, Σ145	2 P119, ?23, Σ144	24 P107, ?5, Σ136
testudines (turtles)	30 P48, ?1, Σ79	30 P52, ?4, Σ86	30 P36, ?1, Σ67	8 P65, ?1, Σ74	8 P88, ?2, Σ98	6 P91, ?2, Σ99	5 P92, ?1, Σ98	0 P70, ?1, Σ71
monotremata (egg-laying mammals)	1 P6, ?0, Σ7	1 P6, ?0, Σ7	1 P6, ?0, Σ7	0 P8, ?0, Σ8	0 P8, ?0, Σ8	2 P6, ?0, Σ8	1 P7, ?0, Σ8	0 P6, ?0, Σ6
metatheria (marsupials)	4 P28, ?0, Σ32	4 P22, ?0, Σ26	4 P22, ?0, Σ26	4 P32, ?0, Σ36	4 P36, ?0, Σ40	4 P35, ?0, Σ39	5 P34, ?0, Σ39	0 P39, ?0, Σ39
eutheria (placentals)	434 P900, ?6, Σ1340	440 P862, ?6, Σ1308	440 P857, ?6, Σ1303	249 P1420, ?11, Σ1680	249 P1504, ?10, Σ1763	171 P1406, ?9, Σ1586	164 P1601, ?9, Σ1774	0 P1596, ?10, Σ1606
tardigrada (water bears)	0 P0, ?4, Σ4	0 P0, ?6, Σ6	0 P0, ?3, Σ3	0 P4, ?0, Σ4	0 P3, ?1, Σ4	0 P2, ?2, Σ4	0 P4, ?0, Σ4	0 P2, ?1, Σ3
onychophora (velvet worms)								
pycnogonida (sea spiders)								
arachnida (spiders)	14 P5, ?22, Σ41	14 P5, ?20, Σ39	13 P5, ?16, Σ34	0 P25, ?13, Σ38	0 P24, ?11, Σ35	1 P16, ?16, Σ33	1 P14, ?2, Σ17	1 P26, ?30, Σ57
xiphosura (horseshoe crabs)	7 P0, ?11, Σ18	7 P0, ?11, Σ18	7 P0, ?11, Σ18	0 P7, ?11, Σ18	0 P7, ?11, Σ18	0 P7, ?4, Σ11	0 P2, ?0, Σ2	0 P7, ?11, Σ18
myriapoda (millipeds)					0 P0, ?1, Σ1			
crustacea	5 P4, ?11, Σ20	5 P4, ?12, Σ21	4 P4, ?8, Σ16	1 P9, ?19, Σ29	1 P6, ?19, Σ26	1 P8, ?13, Σ22	1 P6, ?8, Σ15	1 P7, ?9, Σ17
hexapoda (insects)	49 P53, ?154, Σ256	59 P61, ?198, Σ318	39 P45, ?129, Σ213	5 P110, ?177, Σ292	5 P94, ?156, Σ255	16 P18, ?98, Σ132	2 P36, ?55, Σ93	3 P95, ?236, Σ334
nematomorpha (horsehair worms)								
nematoda (roundworms)				0 P1, ?22, Σ23	0 P0, ?21, Σ21	0 P0, ?4, Σ4	0 P0, ?2, Σ2	0 P0, ?24, Σ24
priapulida (penis worms)		0 P1, ?0, Σ1		0 P1, ?0, Σ1	0 P1, ?0, Σ1	0 P1, ?0, Σ1		0 P1, ?0, Σ1
loricifera								
kinorhyncha (mud dragons)								
chaetognatha (arrow worms)								
bryozoa (moss animals)					0 P0, ?1, Σ1			
entoprocta					0 P0, ?1, Σ1		0 P0, ?1, Σ1	
cycliophora (symbion)								
annelida (segmented worms)	0 P0, ?3, Σ3	0 P0, ?3, Σ3	0 P0, ?3, Σ3	0 P1, ?2, Σ3	0 P1, ?1, Σ2	0 P0, ?3, Σ3	0 P1, ?1, Σ2	0 P1, ?2, Σ3
mollusca	2 P2, ?9, Σ13	2 P2, ?9, Σ13	2 P2, ?5, Σ9	0 P9, ?6, Σ15	0 P4, ?6, Σ10	0 P1, ?15, Σ16	0 P7, ?4, Σ11	0 P8, ?4, Σ12
nemertea (ribbon worms)								
brachiopoda (lamp shells)	0 P1, ?0, Σ1	0 P1, ?0, Σ1	0 P1, ?0, Σ1	0 P1, ?1, Σ2	0 P1, ?0, Σ1	0 P1, ?2, Σ3	1 P0, ?0, Σ1	0 P1, ?0, Σ1
phoroniformea (horseshoe worms)								
gastrotricha (hairybacks)								
platyhelminthes (flatworms)					0 P0, ?1, Σ1			
gnathostomulida (jaw worms)								
micrognathozoa								
rotifera (wheel animals)		0 P1, ?0, Σ1		0 P1, ?0, Σ1	0 P1, ?1, Σ2	0 P1, ?0, Σ1	0 P1, ?0, Σ1	0 P1, ?2, Σ3
orthonectida						0 P0, ?1, Σ1		
dicyemida								

Force topology is enabled!
Branch lengths do not represent real values.
